# Pollenkitt From *Narcissus tazetta*: Evaluation of Its UV‐Protective Potential as a Plant‐Based Biomaterial Source

**DOI:** 10.1002/cbdv.202502122

**Published:** 2025-09-29

**Authors:** Aslıhan Çetinbaş‐Genç, Orçun Toksöz, Özkan Kilin, Nüzhet Cenk Sesal, Giampiero Cai

**Affiliations:** ^1^ Department of Biology Marmara University Kadıköy, Istanbul Turkey; ^2^ Institute of Pure and Applied Sciences Marmara University Istanbul Turkey; ^3^ Department of Life Sciences University of Siena Siena Italy

**Keywords:** antioxidants, natural products, pollen biotechnology, pollenkitt, ultraviolet (UV) protection

## Abstract

Pollenkitt, the lipid‐rich coating of pollen grains, is a promising natural ultraviolet (UV)‐protective source rich in phenolic and flavonoid compounds. This study assessed the UV protection capacity of pollenkitt extracted from *Narcissus tazetta* using six solvents (dH_2_O, acetone, diethyl ether, ethanol, chloroform, and methanol). Methanol extracts showed the highest UV absorbance and sun protection factor (SPF) (17.8), despite 35% lower phenolics than ethanol, suggesting specific compounds like rutin and ferulic acid may drive UV protection. Surprisingly, although the dH_2_O extracts yielded less pollenkitt, they exhibited strong UV absorption SPF (19.1) and antioxidant activity from water‐soluble compounds such as gentisic acid. Absorption, distribution, metabolism, excretion, and toxicity (ADMET) analysis predicted favorable bioavailability and low toxicity for most phenolics, with rosmarinic acid being predicted to possess properties associated with anticarcinogenic potential. However, rutin and naringenin were predicted to exhibit a skin sensitization risk. These findings underscore the potential of pollenkitt, specifically methanol and ethanol extracts, as a safe, natural UV shield for cosmetic, packaging, and coating applications.

## Graphical abstract

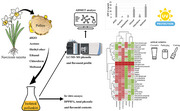

## 1 Introduction

Ultraviolet (UV) radiation had increased globally in the past due to the progressive depletion of the ozone layer [1]. Although recent assessments indicate that the ozone layer is gradually recovering following the Montreal Protocol, prolonged and high levels of UV radiation (as caused by more clear skies following fewer precipitation events) still pose a serious risk to living organisms [2]. Therefore, defense strategies to protect living organisms against the harmful effects of UV rays have been widely investigated [3]. In recent years, research on potential defense and protection strategies using plant‐based materials (known to be versatile, natural, and environmentally friendly) has emerged as promising candidates in various sectors such as cosmetics, coatings, textiles, and food packaging [4]. To this end, the UV‐protective effects of plant extracts from various plant tissues, such as roots, leaves, fruits, and flowers, have been investigated [5], but alternative plant structures as new sources remain largely unexplored.

Pollenkitt (also known as pollen coat or tryphine) is an extracellular layer of pollen grains [6] that is derived from the tapetum and only rarely from different anther components such as the endothecium and microspores. After tapetal cell breakdown, the pollenkitt is deposited on the outermost surface of the pollen grain and fills the spaces and cavities of the exine [7, 8]. Pollenkitt is composed of lipids, carotenoids, phenolics, flavonoids, proteins, and carbohydrates, the proportions of which vary among species [9]. One of the important functions of pollenkitt is to protect pollen grains from UV radiation [9]. Mori et al. [10] found that UV had less effect on the viability of pollen grains with pollenkitt. In addition, Rejón et al. [11] found that UV‐absorbing compounds such as phenolics and flavonoids in pollenkitt prevent UV from reaching the underlying exine, thus protecting the genetic material of pollen grains from UV. On the basis of these studies, we hypothesized that pollenkitt could be a valuable plant component to produce plant‐based UV‐protective materials.

Pollenkitt must be successfully isolated before it can be used as a plant‐based UV protector. The pollenkitt is a hydrophobic layer because it is mostly composed of lipids [9, 12] and can be easily removed by treatment with organic solvents such as cyclohexane, diethyl ether, chloroform, carbon tetrachloride, hexane, heptane, cyclohexane, benzene, diethyl ether, methanol, and ethanol [13, 14, 15]. In addition, pollenkitt isolation frequently involves the use of multiple solvents [16]. Because the amount of raw material used in the production of plant‐based preservatives is important, the amount of pollenkitt isolated is just as important as its successful isolation. Isolated pollenkitt also has a critical UV protection capacity. Antioxidant activity and UV protection capacity are all closely related [17], as antioxidants reduce UV‐induced excessive production of reactive oxygen species. In addition, there is considerable evidence that plant polyphenols such as phenolics and flavonoids, which have antioxidant properties, are the most effective UV‐protective compounds because they act as radical scavengers [18]. The UV protection capacity of the isolated pollenkitt can then be determined by measuring antioxidant activity, phenolic, and flavonoid content. In addition, the profile of phenolic and flavonoid compounds can provide more detailed information on UV protection capacity. Various researchers have emphasized the importance of different chemicals in UV protection, such as caffeic acid and ferulic acid [19], quercetin, luteolin, and catechins [20], resveratrol [21], chlorogenic acid [22], and rutin and quercetin [23]. In addition, information on UV protection capacity can be obtained by measuring UV absorption values as well as the sun protection factor (SPF), which is the most important measurement for determining the efficacy of sunscreen materials [24, 25]. As a result, the UV absorbance and SPF values of isolated pollenkitt can be useful in determining the UV protection capacity of pollenkitt.

The compounds in the plant‐based UV‐protective material are usually evaluated for their physicochemical, medicinal chemistry, excretion, and toxicological properties to assess their potential use as UV‐protective materials. Due to the time and cost constraints of in vitro experiments and the ethical concerns of toxicity testing, computational analyses are often preferred [26]. Low molecular weight (MW) compounds have high absorbency, making them very versatile [27, 28]. Density affects the physical properties, processability, and performance that are critical to the coating and packaging industries. The log *S* and log *P* values define the hydrophilic and lipophilic behavior, which is key to evaluating the effectiveness of UV‐protective materials [29]. p*K*_a_ values provide insight into acidity, improving formulation stability and efficiency [30]. The synthetic accessibility score (SAscore) evaluates the ease of compound synthesis, whereas Lipinski's rule of five predicts bioavailability potential [31, 32]. The natural product score (NPscore) suggests that naturally derived compounds generally have a lower toxicity risk [33]. Plasma clearance (CLplasma) and half‐life (*T*1/2) are critical in determining the duration of action of a compound and the frequency of dosing [34, 35]. From a toxicological perspective, the Ames test assesses potential genotoxic risks, whereas the skin sensitization rule assesses the potential for allergic reactions [36]. The carcinogenicity rule evaluates cancer risk through genotoxic and non‐genotoxic mechanisms. The signal responsive antioxidant response element (SR‐ARE) examines resistance to oxidative stress. The Food and Drug Administration (FDA) Maximum Daily Dose (FDAMDD) establishes toxic dose limits [37]. Nonbiodegradable properties are addressed for environmental persistence and sustainability [38]. Together, these parameters provide critical insight into the design of UV‐protective biomaterials to ensure efficacy, stability, safety, and environmental compatibility. Understanding these properties is essential for tailoring plant‐based UV‐protective materials to meet the needs of various sectors, including cosmetics, textiles, food packaging, and coatings, while promoting sustainability and reducing environmental impact.

This study aims to investigate the potential of pollenkitt, the outermost layer of pollen grains, as a plant‐based UV‐protective material by evaluating its isolation process, UV‐protective capacity, and the physicochemical and toxicological properties of its compounds. We hypothesize that through comparative solvent extraction, including an aqueous extraction, we can identify specific pollenkitt extracts, particularly from methanol and dH_2_O, with superior UV protection and desirable biochemical profiles, thereby validating and optimizing pollenkitt as a sustainable and environmentally friendly source of industrially applicable UV‐protective biomaterials, such as in cosmetics, textiles, food packaging, and coatings.

## 2 Results

### 2.1 Effectiveness of Pollenkitt Isolation and Determination of the Amount of Isolated Pollenkitt

**FIGURE 1:**
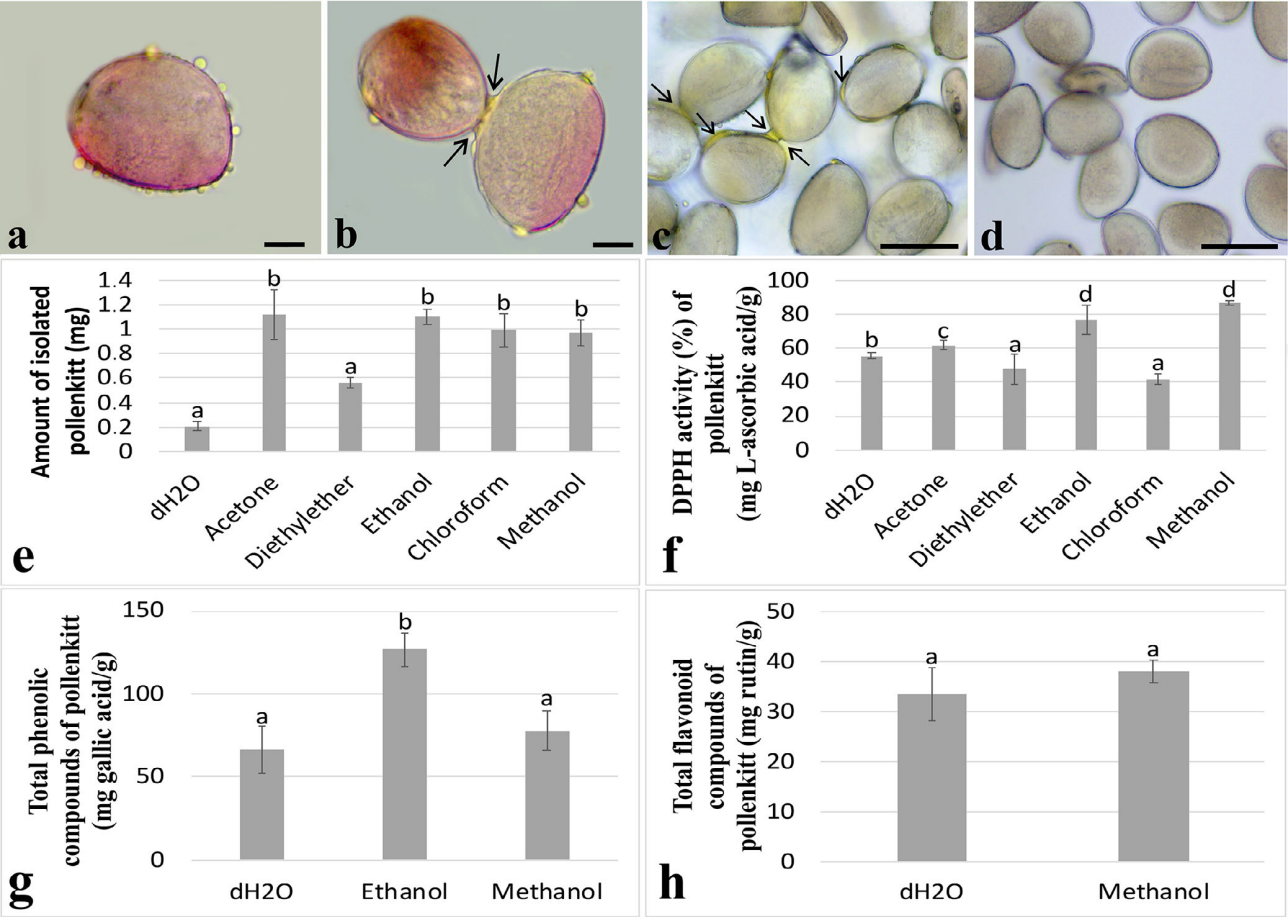
Pollen grains, pollenkitt isolation from the pollen grains, and effects of solvents on biochemical contents of pollenkitt. (a) Pollen grain with pollenkitt. (b) Pollen grains became entangled with pollenkitt. (c) Pollen grains with pollenkitt after dH_2_O treatment. (d) Pollen grains without pollenkitt after solvent treatment. (e) Determination of isolated pollenkitt amount. (f) DPPH% activity of pollenkitt. (g) Total phenolic compounds of pollenkitt. (h) Total flavonoid compounds of pollenkitt. Distinct letters point out the statistically significant differences (*p* < 0.05), and error bars indicate the standard deviations. Bar: 50 µm. DPPH, 2,2‐diphenyl‐1‐picrylhydrazyl.

First, fresh pollen grains were tested for the presence of pollenkitt using a glycerin–gelatin solution containing safranin. According to the observations, the pollenkitt appeared as droplets on the surface of the pollen grains (Figure 1a). In addition, many pollen grains were clumped together with the pollenkitt (Figure 1b). Pollen grains were treated with dH_2_O, acetone, diethyl ether, ethanol, chloroform, and methanol to determine if the pollenkitt was removed. After dH_2_O treatment, the pollenkitt remained visible as yellow in the pollen grains (Figure 1c). However, after treatments with acetone, diethyl ether, ethanol, chloroform, and methanol, the pollenkitt was removed and no lipid droplets were found on the surface of the pollen grains (Figure 1d). According to the amount of pollenkitt on isolates, the most pollenkitt was isolated after acetone, ethanol, chloroform, and methanol treatment with no discernible difference, and the least after diethyl ether and dH_2_O treatment with no significant difference (Figure 1e).

### 2.2 Determination of Total Antioxidant Activity, Total Phenolic, and Flavonoid Contents

The antioxidant activity, total phenolic content, and flavonoid content of isolated pollenkitt were investigated as these are closely related to the UV‐protective capacity of pollenkitt. Antioxidant activity was expressed as percentage inhibition of 2,2‐diphenyl‐1‐picrylhydrazyl (DPPH) radicals, whereas total phenolic and flavonoid contents were calculated on the basis of calibration curves and expressed as mg gallic acid equivalents/g and mg rutin equivalents/g, respectively. Pollenkitt demonstrated the highest DPPH% activity following ethanol (77.29 ± 0.84) and methanol (87.38 ± 0.12) treatments, with no statistically significant difference between the two. Acetone treatment also resulted in relatively high DPPH% activity (61.95 ± 2.79), although it was lower compared to ethanol and methanol. Furthermore, diethyl ether (47.78 ± 9.02) and chloroform (41.85 + 3.39) treatments resulted in the lowest DPPH% activity of pollenkitt, with no statistical difference. After treatments with diethyl ether and chloroform, pollenkitt showed the lowest DPPH% activity of pollenkitt after dH_2_O treatment (Figure 1f). Furthermore, the highest total phenolic content was detected in the ethanol extract (126.76 ± 10.04), followed by methanol (77.84 ± 12.18) and dH_2_O (66.24 ± 14.09). No phenolic content was detected in acetone, diethyl ether, and chloroform extracts. No total phenolic content was detected after treatments with acetone, diethyl ether, and chloroform (Figure 1g). Furthermore, total flavonoid content was determined only in methanol (38.08 ± 2.26) and dH_2_O (33.35 ± 0.52) extracts, with no statistically significant difference between these two groups. Acetone, diethyl ether, ethanol, and chloroform treatments did not result in any detectable total flavonoid content (Figure 1h).

### 2.3 Liquid Chromatography–Tandem Mass Spectrometry (LC–MS/MS) Analysis of Phenolic and Flavonoid Contents

The profiles of phenolic and flavonoid compounds were further analyzed using LC–MS/MS. According to the data, 21 chemicals were found in the pollenkitt extracted with different solvents, of which 12 were phenolic acids and 9 were flavonoids (Table 1). No gallic acid or chlorogenic acid was detected after any solvent treatment. The dH_2_O isolate contained the most gentisic acid, 4‐OH‐benzoic acid, rosmarinic acid, myricetin, luteolin, apigenin, galangin, and chrysin. However, the highest concentrations after dH_2_O isolation were found in the ethanol isolate for gentisic acid and apigenin and in the methanol isolate for 4‐OH‐benzoic acid, rosmarinic acid, myricetin, luteolin, galangin, and chrysin. The dH_2_O isolate did not contain caffeic acid or *trans*‐cinnamic acid. The acetone isolate contained the highest levels of caffeic acid and *p*‐coumaric acid, but no 4‐OH‐benzoic acid and galangin. The diethyl ether isolate contained the most *trans*‐cinnamic acid while having the lowest levels of protocatechualdehyde, ferulic acid, rosmarinic acid, sinapic acid, rutin, and luteolin. In addition, 4‐OH‐benzoic acid, gentisic acid, and caffeic acid were not detected in diethyl ether isolate. Protocatechuic acid, protocatechualdehyde, sinapic acid, resveratrol, naringenin, quercetin, and kaempferol were the most abundant in the ethanol extract, whereas 4‐OH‐benzoic acid was not detected. Of all the isolates tested, the chloroform extract did not have the highest concentration of any phenolic acid or flavonoid compound. However, the chloroform isolate had the lowest levels of protocatechuic acid, salicylic acid, *p*‐coumaric acid, resveratrol, myricetin, naringenin, quercetin, kaempferol, apigenin, and chrysin. In addition, 4‐OH‐benzoic acid and gentisic acid were not detected in the chloroform extract. The methanol isolate contained the highest levels of salicylic acid, ferulic acid, and rutin.

**TABLE 1:** Liquid chromatography–tandem mass spectrometry (LC–MS/MS) analytical results of phenolic and flavonoid compounds in pollenkitt isolated by different solvents (µg/L).

	Compounds	dH_2_O	Acetone	Diethyl ether	Ethanol	Chloroform	Methanol
Phenolic compounds	Gallic acid	—	—	—	—	—	—
	Gentisic acid	36.63	18.74	—	30.23	—	30.11
	Protocatechuic acid	67.91	60.99	19.23	83.60	14.45	69.86
	Protocatechualdehyde	7.57	6.90	5.13	12.86	5.81	8.04
	4‐OH‐Benzoic acid	47.35	—	—	—	—	30.40
	Salicylic acid	379.15	365.9	313.51	481.44	22.09	523.90
	Chlorogenic acid	—	—	—	—	—	—
	Caffeic acid	—	24.46	—	19.76	11.11	21.36
	*p*‐Coumaric acid	236.98	806.33	184.68	315.88	20.67	212.01
	Ferulic acid	33.34	61.90	4.61	69.73	6.39	129.87
	Rosmarinic acid	589.00	288.33	135.76	384.69	195.84	491.88
	Sinapic acid	50.26	44.66	25.98	76.26	44.59	52.52
	*Trans*‐cinnamic acid	—	33.37	47.27	21.97	23.21	43.73
	Resveratrol	327.85	477.27	103.43	554.62	58.28	374.34
Flavonoids	Rutin	141.47	70.08	11.92	143.55	23.75	324.97
	Myricetin	1547.57	381.74	175.49	1071.76	140.71	1306.39
	Naringenin	10.63	9.99	11.26	12.16	7.51	11.08
	Quercetin	618.67	601.19	374.46	1125.76	147.28	939.98
	Luteolin	345.05	50.25	47.82	96.70	58.57	154.66
	Kaempferol	7189.14	38 705.91	23 884.56	48 236.59	5562.05	36 949.38
	Apigenin	71.11	25.09	16.60	28.11	15.08	27.44
	Galangin	255.50	—	2.85	9.22	5.31	34.50
	Chrysin	70.61	3.27	4.30	7.30	1.28	10.92

### 2.4 Determination of UV‐A/UV‐B Absorbance and SPF Value

**FIGURE 2:**
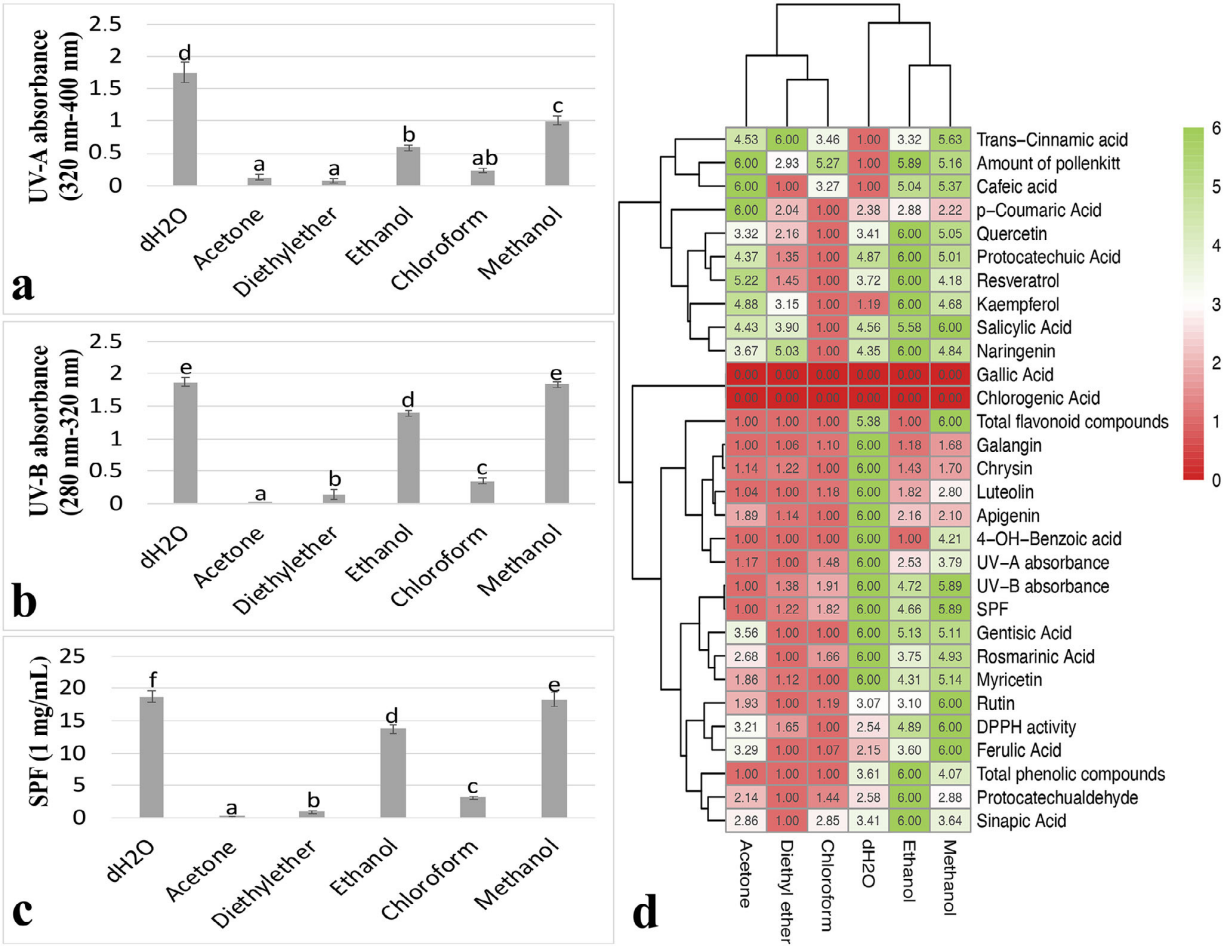
UV absorbance, UV protection capacities of pollenkitt, and heat‐map analysis of solvents. (a) UV‐A absorbance of pollenkitt. (b) UV‐B absorbance of pollenkitt. (c) SPF of pollenkitt. (d) Heat‐map analysis of solvents. Distinct letters point out the statistically significant differences (*p* < 0.05), and error bars indicate the standard deviations. DPPH, 2,2‐diphenyl‐1‐picrylhydrazyl; SPF, sun protection factor; UV, ultraviolet.

UV absorptions of pollenkitt isolated with different solvents were measured at wavelengths ranging from 280 to 400 nm. UV‐B and UV‐A absorbance values were determined in the following descending order: dH_2_O, methanol, ethanol, chloroform, diethyl ether, and acetone (Figure 2a,b). In order to determine the UV protection capacities, the SPF values of pollenkitt isolated with different solvents were calculated. According to the calculations, the dH_2_O isolate had the highest SPF value and the acetone isolate had the lowest SPF value. After dH_2_O, the methanol and ethanol isolates showed the highest values. In addition, diethyl ether and chloroform isolates had the lowest values after acetone (Figure 2c).

### 2.5 Heat‐Map Analysis

A heatmap was created to collectively evaluate the parameters of the solvents and determine the most appropriate solvent. The columns with the strongest green color represent the groups with the highest values, whereas the columns with the strongest red color represent the groups with the lowest rates of change. Methanol and ethanol were assigned to the same sub‐cluster, which then merged with dH_2_O in a higher level cluster. Similarly, chloroform and diethyl ether were assigned to the same sub‐cluster, which then merged with acetone to form a higher level cluster. In addition, a ranking was determined by calculating the total value of the cells in each column. The ranking showed that methanol, ethanol, and dH_2_O had the highest values, whereas chloroform, diethyl ether, and acetone had the lowest values (Figure 2d).

### 2.6 Absorption, Distribution, Metabolism, Excretion, and Toxicity (ADMET) Analysis

The ADMET scores, which include physicochemical properties, medicinal chemistry, and excretion characteristics, were used to evaluate the applicability of the phenolic and flavonoid compounds identified in the pollenkitt by LC–MS/MS analyses in relevant industries. The results are shown in Table 2. The MWs of the phenolic compounds ranged from 138.03 to 360.08, whereas the MWs of the flavonoid compounds ranged from 254.06 to 610.15. The density values of all molecules were determined to be between 0.9 and 1.1. All phenolic compounds tested had log *S* values between −4 and 0.5 log mol/L, which is considered optimal. However, among the flavonoid compounds, only chrysin, apigenin, luteolin, and naringenin were found to be outside of this optimal range. On the basis of log *P* values, gallic acid and protocatechuic acid among the phenolic compounds and rutin and myricetin among the flavonoid compounds were found to have a high tendency for lipophilicity. The p*K*_a_ (acid) values of protocatechualdehyde and resveratrol among the phenolic substances were 7.27 and 9.62, respectively, whereas the other molecules were in the range of 3–5, and the p*K*_a_ (acid) values of the flavonoid contents were in the range of 4–8. All of the p*K*_a_ (basic) values were in the range of 1–5. The SAscore of all phenolic and flavonoid compounds was determined to be in the “easy” category. According to Lipinski's rule evaluation, all phenolic compounds were classified as “accepted” as drug‐like substances. Rutin was the only flavonoid compound classified as “rejected,” with all other flavonoids classified as “accepted.” According to the NPscore analysis, the phenolic compounds with the highest scores were chlorogenic acid (2.246), caffeic acid (1.124), and rosmarinic acid (1.128). In the analysis of flavonoid compounds, rutin had the highest NPscore (2.015), whereas all other flavonoid compounds had NPscore values greater than 1. The CLplasma values showed that the phenolic compounds 4‐hydroxybenzoic acid, salicylic acid, and chlorogenic acid, as well as the flavonoid compounds rutin, galangin, and chrysin, had a low clearance. All other phenolic and flavonoid compounds showed moderate clearance. In addition, among the flavonoid compounds, chrysin had an ultra‐short half‐life with a *T*1/2 value of 0.967, whereas rutin had a moderate half‐life (*T*1/2: 4.616). The remaining flavonoid and phenolic compounds were found to have short half‐lives.

**TABLE 2:** Absorption, distribution, metabolism, excretion, and toxicity (ADMET) scores of phenolic and flavonoid compounds based on their physicochemical, medicinal, and excretion properties.

		Physicochemical property						Medicinal chemistry			Excretion	
	Compounds	MW	Density	Log *S*	Log *P*	p*K*_a_ (acid)	p*K*_a_ (base)	SAscore	Lipinski rule	NPscore	CLplasma	*T*1/2
Phenolic compounds	Gallic acid	170.02	1.10	−1.55	0.69	4.49	2.58	Easy	+	0.98	5.40	2.2
	Gentisic acid	154.03	1.05	−2.03	1.39	4.16	4.22	Easy	+	0.73	13.91	1.72
	Protocatechuic acid	154.03	1.05	−1.69	1.00	4.15	2.02	Easy	+	0.73	4.94	2.25
	Protocatechualdehyde	152.05	0.98	−1.77	1.12	7.27	4.14	Easy	+	0.85	10.28	1.52
	4‐OH‐Benzoic acid	138.03	1.00	−1.77	1.23	4.27	2.55	Easy	+	0.37	3.45	1.66
	Salicylic acid	138.03	1.00	−1.99	2.26	3.81	2.59	Easy	+	0.13	2.66	1.31
	Chlorogenic acid	354.1	1.06	−2.95	1.03	4.22	4.10	Easy	+	2.24	3.34	2.75
	Caffeic acid	180.04	1.01	−1.83	1.19	4.47	2.80	Easy	+	1.12	14.35	2.07
	*p*‐Coumaric acid	164.05	0.97	−2.11	1.44	4.57	2.98	Easy	+	0.84	7.54	1.57
	Ferulic acid	194.06	0.99	−2.36	1.64	4.36	3.36	Easy	+	0.92	8.35	1.69
	Rosmarinic acid	360.08	1.03	−3.03	2.00	5.38	3.13	Easy	+	1.12	13.23	1.90
	Sinapic acid	224.07	1.01	−2.54	1.68	4.37	4.89	Easy	+	0.76	5.72	2.20
	*Trans*‐cinnamic acid	148.05	0.92	−3.35	2.61	4.30	1.70	Easy	+	0.40	6.16	1.88
	Resveratrol	228.08	0.94	−3.60	2.89	9.62	4.53	Easy	+	0.75	9.03	1.45
Flavonoid	Rutin	610.15	1.10	−2.39	0.98	4.57	5.32	Easy	−	2.01	1.61	4.61
	Myricetin	318.04	1.09	−3.44	1.11	6.26	1.43	Easy	+	1.69	6.77	1.62
	Naringenin	272.07	1.01	−4.02	2.59	8.97	5.13	Easy	+	1.76	6.89	1.31
	Quercetin	302.04	1.06	−3.72	1.44	6.40	2.08	Easy	+	1.70	8.28	1.58
	Luteolin	286.05	1.04	−4.01	2.24	7.94	3.19	Easy	+	1.49	8.48	1.37
	Kaempferol	286.05	1.04	−3.64	1.96	6.81	3.64	Easy	+	1.54	5.69	1.38
	Apigenin	270.05	1.01	−4.21	2.98	8.40	3.66	Easy	+	1.35	5.93	1.20
	Galangin	270.05	1.01	−3.19	2.22	5.84	3.60	Easy	+	1.42	4.63	1.05
	Chrysin	254.06	0.99	−4.88	3.6	8.90	3.38	Easy	+	1.22	4.66	0.96

Abbreviation: MW, molecular weight; NPscore, natural product score; SAscore, synthetic accessibility score.

The toxicity parameters of the phenolic and flavonoid compounds found in the pollenkitt were determined using ADMET scoring, and the results are presented in Table 3. The flavonoid molecules had moderate mutagenic activity, as determined by the Ames toxicity parameters, with values ranging from 0.546 to 0.796. Among the phenolic compounds, resveratrol, rosmarinic acid, protocatechualdehyde, and gentisic acid were found to have moderate mutagenic activity, whereas the remaining phenolic compounds had low mutagenic activity, with values ranging from 0 to 0.3. Rosmarinic acid and resveratrol also exceeded the recommended FDAMDD value, with 0.924 and 0.882, respectively. In addition, all flavonoid compounds except rutin scored higher than the FDAMDD (scores above 0.7), indicating that they may be highly toxic. Furthermore, the phenolic acids 4‐hydroxybenzoic acid, salicylic acid, and sinapic acid as well as the flavonoids galangin and chrysin showed a moderate skin sensitization potential with values ranging from 0.4 to 0.5. All phenolic compounds were found to have low to moderate carcinogenic activity, with values ranging from 0 to 0.5. Among the flavonoids, kaempferol, apigenin, galangin, and chrysin had values greater than 0.7, indicating carcinogenic potential. According to the SR‐ARE parameter analysis, most phenolic compounds and only rutin among flavonoids showed excellent results, with values ranging from 0 to 0.1 (− −), 0.1 to 0.3 (−), and 0.3 to 0.5 (−). According to the Genotoxic Carcinogenicity Mutagenicity Rule, only chlorogenic acid and rosmarinic acid received a value of 1. In contrast, the non‐genotoxic carcinogenicity rule assigned a value of 1 to the following compounds: chlorogenic acid, caffeic acid, *p*‐coumaric acid, ferulic acid, rosmarinic acid, sinapic acid, and *trans*‐cinnamic acid. All compounds, except for 4‐hydroxybenzoic acid and salicylic acid (both with a value of 0), had moderate to high skin irritation potential, with values ranging from 2 to 8. All compounds had low nonbiodegradability potentials (values ranging from 0 to 2).

**TABLE 3:** Absorption, distribution, metabolism, excretion, and toxicity (ADMET) scores of phenolic and flavonoid compounds based on their toxicity parameters.

	Compounds	Ames toxicity	FDAMDD	Skin sensitization	Carcinogenicity	SR‐ARE	Genotoxic carcinogenicity mutagenicity rule	Non‐genotoxic carcinogenicity rule	Skin sensitization rule	Nonbiodegradable
Phenolic compounds	Gallic acid	0.48	0.20	0.99	0.21	+ + +	0	0	7	1
	Gentisic acid	0.40	0.19	0.64	0.3	+ + +	0	0	4	1
	Protocatechuic acid	0.37	0.17	0.90	0.33	+	0	0	5	1
	Protocatechualdehyde	0.46	0.23	0.8	0.57	− −	0	0	6	0
	4‐OH‐Benzoic acid	0.27	0.13	0.41	0.42	−	0	0	0	0
	Salicylic acid	0.31	0.11	0.43	0.28	−	0	0	0	0
	Chlorogenic acid	0.38	0.41	0.98	0.22	−	1	1	8	1
	Caffeic acid	0.27	0.30	0.97	0.17	−	0	1	7	1
	*p*‐Coumaric acid	0.21	0.27	0.78	0.18	−	0	1	2	0
	Ferulic acid	0.25	0.19	0.74	0.24	−	0	1	6	0
	Rosmarinic acid	0.54	0.92	1	0.07	−	1	1	8	1
	Sinapic acid	0.21	0.29	0.43	0.39	−	0	1	6	0
	*Trans*‐cinnamic acid	0.21	0.22	0.79	0.12	− −	0	1	2	0
	Resveratrol	0.60	0.82	0.94	0.43	+ + +	0	0	5	1
Flavonoids	Rutin	0.75	0.13	0.99	0.04	−	0	0	8	2
	Myricetin	0.65	0.86	0.99	0.50	+ +	0	0	8	1
	Naringenin	0.70	0.74	0.74	0.59	+ + +	0	0	6	1
	Quercetin	0.58	0.78	0.89	0.6	+ + +	0	0	8	1
	Luteolin	0.79	0.88	0.92	0.68	+ + +	0	0	7	1
	Kaempferol	0.54	0.80	0.62	0.71	+ + +	0	0	4	1
	Apigenin	0.61	0.88	0.64	0.79	+ + +	0	0	3	1
	Galangin	0.56	0.75	0.57	0.70	+ + +	0	0	4	1
	Chrysin	0.62	0.84	0.59	0.78	+ + +	0	0	3	1

Abbreviations: FDAMDD, Food and Drug Administration Maximum Daily Dose; SR‐ARE, signal responsive antioxidant response element.

## 3 Discussion

The UV‐protective properties of pollenkitt, found in the pollen grains of many angiosperm species, are well known; however, its UV‐protective capacity has never been directly tested with concrete data in previous studies [9]. Pollenkitt, a hydrophobic layer, has been reported to be easily isolated from pollen grains of different plant species using different organic solvents for different purposes [12]. Dobson [39] used benzene to remove the pollenkitt layer and characterized the lipid compositions of pollenkitt from 69 angiosperm species. Cyclohexane has been used for pollen kit isolation in several studies, including the identification of pollenkitt‐specific oleosins in *Brassica oleracea* [40], the extraction of tapetal oleosin‐like fusion protein in *Brassica carinata* [41], the analysis of pollenkitt proteins in *Cynodon dactylon* [42], and the characterization of pollenkitt biomolecules in *Helianthus annuus* [15]. Additionally, diethyl ether and chloroform were used for pollenkitt extraction and fatty acid analysis in *Oryza sativa* [43]. Chichiriccò et al. [14] used carbon disulfide to study the lipid components of pollenkitt in *Crocus vernus* and *Narcissus poeticus*. Various solvent combinations have also been used extensively for pollenkitt isolation. For example, Lin et al. [16] used chloroform and methanol to extract pollenkitt from *Taraxacum officinale* and *H. annuus* pollen and determined the amount of pollen cement. However, most of these studies have focused on the analysis of the biomolecules within the isolated pollenkitt, with little emphasis on the volume of pollenkitt extracted by different solvents.

In this study, we hypothesized that the isolated pollenkitt may hold great promise for the development of plant‐based UV‐protective materials. Similar plant‐based strategies have recently been employed in textile applications, such as the incorporation of chitosan‐enriched milk thistle extract‐loaded liposomes onto nonwoven cotton fabrics [44], showing strong photoprotective and multifunctional properties. Since the amount of raw material is a critical factor in biotech and industrial product development processes, we first identified the solvent that yielded the highest amount of pollenkitt isolate. According to our results, acetone, ethanol, chloroform, and methanol yielded the highest amount of isolated pollenkitt, with no statistically significant differences among them. Although there was no statistically significant difference, dH_2_O and diethyl ether produced the least amount of pollenkitt. This variation between solvents is thought to be due to differences in their polarity values. It is well known that solvents with different polarities can extract lipid fractions from lipoidal layers, such as pollenkitt, in different ways.

In industrial biotechnology, raw material quantity and quality are critical parameters in the product development process. To evaluate the UV‐protective capacity of the isolated pollenkitt, we investigated its antioxidant activity, total phenolic content, and flavonoid content. In fact, researchers have suggested that the high antioxidant activity, phenolic, and flavonoid content of plant components used as UV filters contribute to UV filtration [45]. In addition, the pollenkitt layer is known to be rich in antioxidant activity, phenolics, and flavonoids [46]. Our results showed that the ethanol and methanol extracts had the highest antioxidant activity, with no statistically significant difference between them. This result is consistent with previous studies reporting that major antioxidant compounds are highly soluble in ethanol and methanol [47]. A comparison of ethanol and methanol extracts of *Daemonorops acehensis* resin showed that methanol extracts exhibited higher antioxidant activity than ethanol extracts [45]. It is interesting to note that the dH_2_O extract had significantly higher antioxidant activity than the diethyl ether and chloroform extracts. Similarly, Sari et al. [45] found that the dH_2_O extract of *Gyrinops versteegii* leaves had significantly high antioxidant activity, comparable to the 100% ethanol extract, which gave the best results.

Phenols and flavonoids, which contain double bonds or aromatic rings, can absorb UV radiation in the 200–400 nm range, making them ideal for sunscreen formulations [48]. Our study found that total phenolic content was present only in dH_2_O, ethanol, and methanol extracts, with methanol showing the highest content. The total flavonoid content was observed exclusively in dH_2_O and methanol extracts with no significant difference between them. This variation is likely due to factors such as plant species, tissue type, distribution of phytochemicals within the tissue, and solvent polarity. Studies have shown similar trends in the UV‐protective activity of isolates with different structures. For example, no phenolic content was detected in ethanol extracts of *D. acehensis* resin [45], whereas methanol extracts of *Symphytum officinale* seeds had significantly higher flavonoid content compared to ethanol extracts [49]. Consistent with the antioxidant activity results, phenolics and flavonoids were present in dH_2_O extracts but not in some other solvents, suggesting that low MW phenolic compounds or specific flavonoids with antioxidant properties that are more soluble in dH_2_O may play a critical role in UV protection.

Numerous studies have shown that various phenolic compounds, including protocatechuic acid, protocatechualdehyde, and sinapic acid, have anti‐UV potential due to their antioxidant and tyrosinase inhibitory activities [50]. In our case, these compounds were mainly found in ethanol extracts. Quercetin and kaempferol, known for their strong UV‐absorbing capacity, were found in the highest concentrations in ethanolic extracts by Emiliani et al. [51]. In addition, rutin, quercetin, and naringenin are often reported for their photoprotective properties, with rutin being most concentrated in methanol extracts and quercetin and naringenin in ethanol extracts [5]. These results emphasize the importance of solvent selection in the isolation process as it significantly affects the extraction of phenolic and flavonoid compounds.

Our results highlight the importance of aqueous solvents, with the highest UV‐A absorbance observed in H_2_O, followed by methanol and ethanol. For UV‐B absorbance, dH_2_O and methanol showed the highest absorbance with no significant difference, followed by ethanol. Similarly, the highest SPF value was found in the dH_2_O extract, followed by methanol and ethanol. Although dH_2_O did not provide a high amount of pollenkitt, it had strong UV absorption and a high SPF. This suggests that low MW phenolic compounds such as gentisic acid and antioxidant flavonoids such as luteolin and apigenin, which are soluble in dH_2_O, may contribute to UV protection. LC–MS/MS results further supported this hypothesis, showing the highest concentrations of gentisic acid, luteolin, and apigenin in dH_2_O extracts. However, we acknowledge that the low predicted aqueous solubility of luteolin and apigenin (log *S* ≈ −4) may restrict their individual contributions. Therefore, the strong UV absorbance and SPF values of the aqueous extract are more likely due to a combination of highly water‐soluble phenolics, such as gentisic acid, and possible synergistic effects within the pollenkitt matrix. In addition, it should be noted that residual proteins, glycoproteins, polysaccharides, or colloidal components within the pollenkitt matrix could also contribute to the apparent UV absorbance through light‐scattering effects. Although not directly assessed in the present study, this possibility is acknowledged as a limitation and may be addressed in future research.

Both ethanol and methanol extracts showed high antioxidant activity. Although the ethanol extract had a higher total phenolic content, it lacked total flavonoid content. LC–MS/MS analyses revealed the highest concentrations of various phenolic and flavonoid compounds in the ethanol extract. However, the methanol extract showed greater UV absorption and a higher SPF value than the ethanol extract. This suggests that higher concentrations of compounds such as rutin, ferulic acid, and salicylic acid in the methanol extract may have contributed to its superior UV protection. Various phenolic and flavonoid compounds have been found to have different absorption characteristics at different UV wavelengths [52]. The synergistic effects of the compounds in the methanol extract may have enhanced its UV‐absorbing performance.

According to FDA recommendations, a formulation can be classified as a sunscreen if it has an SPF greater than 2, but an SPF of 15 or higher is recommended for adequate protection. In our study, the SPF values of dH_2_O and methanol extracts were above 15, whereas the ethanol extract exceeded 10. These results suggest that the pollen layer has significant potential for the development of plant‐based UV‐protective materials. Notably, 30 mg of pollen‐derived pollenkitt showed SPF values ranging from 13 to 19 in various extracts, highlighting its promise compared to other plant extracts with SPF values ranging from 0.97 to 7.38 [53]. The dependence of SPF values on plant species, tissue type, solvent, and isolation protocol has been described in detail by Li et al. [4], but no information on pollen or pollen‐derived layers is included. This gap in the literature underscores the novelty and significance of our findings, which could advance plant‐based UV‐protective formulations.

Our heat‐map analysis suggested that methanol and ethanol extracts would produce better results. However, for commercial applications, ethanol extraction is preferred to methanol because it is less flammable, toxic, and odorous.

The use of isolated compounds in industries, such as cosmetics, coatings, textiles, and food packaging, may pose therapeutic and toxicological risks that affect their applicability. Given the time and cost challenges of in vitro experimentation, novel computational models can accelerate risk assessment through predictive analysis. This study used ADMET analysis to evaluate the physicochemical properties and toxicological risks of compounds identified via LC/MS–MS analysis. Low MW compounds are critical for absorption and distribution because they more easily penetrate biological membranes. All identified phenolic and flavonoid compounds, except rutin, had low MWs. Rutin (MW: 610.15) violates Lipinski's rules due to its high MW, which was predicted to indicate low oral bioavailability [54].

According to the SAscore, compounds with a score below 6 are easier to synthesize, and all isolated phenolic and flavonoid compounds in our study had SAscore values below 6, suggesting a predicted ease of synthesis [55]. Most compounds were predicted to meet Lipinski's rule of five for predicting oral bioavailability, except for rutin, which has a high MW (610.15) and was predicted to have low oral bioavailability [54]. The NPscore, which assesses how closely a molecule resembles natural products, indicated that all isolated molecules had predicted values within the expected range [33]. Additionally, most phenolic and flavonoid compounds showed predicted moderate plasma clearance and short half‐lives (*T*1/2 values ranging from 1.05 to 2.75 h). Although these systemic PK parameters, such as plasma clearance and half‐lives, are not directly relevant to cosmetic, packaging, and coating applications, they were considered complementary ADMET outputs to provide a broader view of the compounds’ safety and stability profiles and to support their potential applicability as biomaterials. For such applications, photostability, local retention, and formulation compatibility are more critical factors; these were not measured in this study but represent an important direction for future research.

The Ames toxicity test is critical for screening compounds for potential carcinogenicity. In our study, resveratrol (0.60), rutin (0.75), luteolin (0.79), and naringenin (0.70) had Ames toxicity scores based on in silico ADMET; these values indicate a model‐predicted mutagenicity risk and warrant experimental validation. Conversely, gallic acid (0.48), caffeic acid (0.27), and sinapic acid (0.21) had lower Ames toxicity scores, which are consistent with a lower mutagenicity risk in this model. The FDAMDD assesses the toxic dose threshold of chemicals. All phenolic compounds had acceptable FDAMDD values, except resveratrol and rosmarinic acid, whose values exceeded the model threshold, indicating a potential toxicity concern based on this prediction [37]. Most flavonoid compounds showed low safety profile scores in the model, suggesting a potential risk of exceeding safe daily intake levels. Skin sensitization, important in various industries, evaluates the potential for allergic skin reactions [26]. Except for 4‐OH‐benzoic acid and salicylic acid, all compounds were predicted to have a moderate to high skin sensitivity risk.

Carcinogenicity assessments are essential for estimating cancer risk from long‐term exposure to chemicals. ADMETLab 3.0 data classify carcinogenicity scores as follows: 0–0.3 (low toxicity), 0.3–0.7 (moderate toxicity), and 0.7–1.0 (high toxicity). In our study, phenolic compounds had carcinogenicity values in the low to moderate range, whereas flavonoid compounds had values in the moderate to high range according to the ADMETLab 3.0 model. Rosmarinic acid had the lowest carcinogenicity value (0.072), which is consistent with reports of its potential anticancer properties [56]. Most phenolic compounds, with the exception of gallic acid, gentisic acid, and resveratrol, showed model‐predicted SR‐ARE activation, which is associated with oxidative stress mitigation. Among flavonoids, only rutin exhibited model‐predicted SR‐ARE activation, which may be associated with oxidative stress regulation.

Toxiphor Rules predict the toxicity potential of molecules based on genotoxic and non‐genotoxic carcinogenicity, skin sensitization, and biodegradability. Our study found that only chlorogenic acid and rosmarinic acid showed model‐predicted genotoxic risks. According to the in silico assessment, seven phenolic compounds, including chlorogenic acid and rosmarinic acid, showed predicted non‐genotoxic carcinogenicity risks. The model did not predict genotoxic or non‐genotoxic carcinogenicity risks for any of the flavonoid compounds. All compounds, except 4‐OH‐benzoic acid and salicylic acid, were predicted to have a high risk of skin sensitization. According to the in silico assessment, only rutin showed low biodegradability, whereas the other compounds were classified as biodegradable. These findings highlight the diverse toxicity profiles of phenolic and flavonoid compounds, emphasizing the need for comprehensive safety assessments.

## 4 Conclusion

Our results show that the solvent used has a significant influence on both the amount and the biochemical composition of the isolated pollenkitt, which determines its UV‐protective capacity. Ethanol showed the highest isolation efficiency, whereas methanol extracts showed superior UV absorbance and SPF values, likely due to bioactive compounds such as rutin and ferulic acid. Surprisingly, although dH_2_O did not fully isolate pollenkitt, it still showed high UV absorbance and antioxidant activity, suggesting its potential for selective extraction of UV‐protective compounds. According to the in silico ADMET analysis, most phenolic compounds, particularly rosmarinic acid, showed predictions consistent with favorable bioavailability and low toxicity, whereas flavonoids exhibited higher skin sensitization potential, supporting the importance of comprehensive safety evaluation. These results support the use of methanol and ethanol for pollen extraction in plant‐based UV protection formulations. The unexpected efficiency of dH_2_O also suggests the potential for less invasive extraction methods. Further research should optimize extraction techniques and assess long‐term safety for industrial applications in pharmaceuticals, cosmetics, textiles, and food packaging.

## 5 Experimental Section

### 5.1 Pollen Material and Pollenkitt Isolation

This study used *Narcissus tazetta* pollen grains, which contain a high amount of pollenkitt [14]. Pollen was collected from plants in the Akçakoca/Düzce region (Turkey). They were dehydrated and stored at −20°C until use. Fresh pollen grains were mounted on glycerin–gelatin‐coated slides, supplemented with safranin, and examined under a light microscope to detect the presence of the pollenkitt.

Six different solvents were used to remove pollenkitt from pollen grains: dH_2_O, acetone, diethyl ether, ethanol, chloroform, and methanol in their absolute forms. We did not dilute the organic solvents to test their full effectiveness [14, 15]. Following the pollen rehydration process, 30 mg of pollen grains were resuspended in 1 mL of solvent in a tube, vortexed for 1 min, and centrifuged at 5000 *g* for 5 min. Pellets were examined under a light microscope to determine if pollenkitt could be separated from pollen grains using different solvents. The supernatants were used as a pollenkitt source to determine the total antioxidant activity, total phenolic, and flavonoid content, for LC–MS/MS analysis, UV‐absorbing capacities, and SPF values.

### 5.2 Determination of the Amount of Isolated Pollenkitt

Before the isolation procedure, the weights of all tubes were precisely measured using an analytical balance and systematically documented. Following the pollenkitt isolation, the supernatants were kept at room temperature for 30 min in a vacuum incubator to evaporate the solvents and obtain pure pollenkitt. The weights of the tubes containing purified pollenkitt were determined using a precision balance and recorded. The amount of pollenkitt extracted from 30 mg of pollen using different solvents was calculated by subtracting the initial tube weight measurements from the final tube weight measurements.

### 5.3 In Vitro Determination of Total Antioxidant Activity, Total Phenolic, Total Flavonoid Contents

After the solvents had evaporated, pure pollenkitt was mixed with the solvents to a final concentration of 1 mg/mL. To determine the DPPH radical scavenging activity, 50 µL pollenkitt solution was mixed with 50 µL DPPH solution and incubated in the dark for 30 min. The absorbances of pollenkitt solutions were spectrophotometrically measured at 517 nm [57]. Each group's solvent served as a blank, whereas l‐ascorbic acid was used as a positive control. The DPPH% radical scavenging activity was calculated using the formula proposed by [58], as given in the following formula:

$$\mathrm{DPPH}\%=\left(\frac{\mathrm{Abs}517\mathrm{blank}-\mathrm{Abs}517\mathrm{treatment}}{\mathrm{Abs}517\mathrm{blank}}\right)\times 100$$

To determine the total phenolic content, 50 µL pollenkitt solutions were mixed with 50 µL of Folin–Ciocalteu reagent and 50 µL of 7.5% Na_2_CO_3_. The absorbances of the pollenkitt solutions were spectrophotometrically measured at 750 nm after 1 h incubation in the dark [58]. Each group's own solvent served as a blank for pollenkitt. Gallic acid at a stock concentration of 1 mg/mL was used to make the calibration curve. The total flavonoid content was determined by mixing 50 µL pollenkitt solutions with 50 µL of 2% AlCl_3_ and 50 µL of sodium citrate solutions. After 15 min incubation in the dark, the absorbances of the pollenkitt solutions were spectrophotometrically measured at 435 nm [58]. Each group's own solvent was used as a blank. Rutin was used to create calibration curves.

### 5.4 LC–MS/MS Analysis of Phenolic and Flavonoid Contents

The pure pollenkitt was combined with 100% ethanol to achieve a final concentration of 1 mg/mL for LC–MS/MS analysis. A 50 µL aliquot of the pollenkit sample was combined with the internal standard solution and then treated with the extraction reagent. After centrifugation at 3600 *g* for 5 min, the supernatant was used for chromatography. To ensure accurate detection in each analysis, the mass spectrometric parameters of the standard and sample ions were optimized.

### 5.5 In Vitro Determination of UV‐A and UV‐B Absorbance and SPF Value

Following the pollenkitt isolation procedure, supernatants were kept at room temperature in a vacuum incubator for 30 min to evaporate the solvents and obtain pure pollenkitt. The pollenkitt was combined with its solvents to achieve a final concentration of 1 mg/mL. Prior to analysis, extracts were centrifuged at 5000 *g* for 10 min and filtered through 0.22 µm syringe filters to remove particulates, thereby minimizing turbidity. Solvent‐specific blanks were also recorded and used for baseline correction, minimizing the impact of potential scattering artifacts. The absorbances of pollenkitt solutions were measured every 5 nm between 280 and 400 nm with a UV spectrophotometer. The solvents from each group were used as controls. SPF values were calculated spectrophotometrically according to Mansur's equation [59] using absorbance values between 290 and 320 nm, as given in the following formula:

$$\mathrm{SPF}=\mathrm{CF}\times \sum_{290}^{320}\left[\mathrm{EE}\left(\lambda \right)\times \;I\left(\lambda \right)\times \mathrm{Abs}\left(\lambda \right)\right]$$

In the formula, CF is the correction factor (=10), EE(*λ*) is the erythemal effect, *I*(*λ*) is the solar intensity, and Abs(*λ*) is the sample absorbance at wavelength *λ*. The EE × *I* constants were obtained from Sayre et al. [60] and are shown in Table 4. Absorbance values between 320 and 400 nm were measured only for evaluating UV‐A protective capacity.

**TABLE 4:** Normalized product function used in the calculation of sun protection factor (SPF) [60].

***λ***	290	295	300	305	310	315	320	Total = 1
**EE × *I***	0.0150	0.0817	0.2874	0.3278	0.1864	0.0839	0.0180	

### 5.6 Statistical and Computational Analysis

Statistical analysis was performed using SPSS 16.0 software, and data were subjected to one‐way ANOVA with a *p* value threshold of 0.05. To analyze and visualize the data, a heatmap was generated using the “pheatmap” function in the R programming language and the RStudio integrated development environment. The ADMET analysis was performed using ADMETlab3.0 (https://admetlab3.scbdd.com/) to evaluate physicochemical properties, medicinal chemistry, excretion, and toxicity using the canonical SMILES formulas of phenolic and flavonoid compounds isolated from pollenkit (Supporting Information).

## Author Contributions

**Aslıhan Çetinbaş‐Genç**: conceptualization, investigation, interpretation, writing original draft. **Orçun Toksöz**: conceptualization, investigation, interpretation, writing original draft. **Özkan Kilin**: data curation, investigation. **Nüzhet Cenk Sesal**: data curation, funding acquisition. **Giampiero Cai**: conceptualization, interpretation, supervision, writing original draft, editing. All authors read and approved the final manuscript.

## Conflicts of Interest

The authors declare no conflicts of interest.

## Data Availability

Data will be made available on request.
